# Multi-Armed Bandit-Based User Network Node Selection

**DOI:** 10.3390/s24134104

**Published:** 2024-06-24

**Authors:** Qinyan Gao, Zhidong Xie

**Affiliations:** 1 National Innovation Institute of Defense Technology, Academy of Military Science, Beijing 100010, China; gaoqinyan2020@163.com; 2 Intelligent Game and Decision Laboratory, Academy of Military Science, Beijing 100091, China; 3 Chinese People’s Liberation Army 32806 Unit, Academy of Military Science, Beijing 100091, China

**Keywords:** online learning, SAGIN, ε-greedy, UCB, Thompson sampling, dynamic variance sampling, MAB

## Abstract

In the scenario of an integrated space–air–ground emergency communication network, users encounter the challenge of rapidly identifying the optimal network node amidst the uncertainty and stochastic fluctuations of network states. This study introduces a Multi-Armed Bandit (MAB) model and proposes an optimization algorithm leveraging dynamic variance sampling (DVS). The algorithm posits that the prior distribution of each node’s network state conforms to a normal distribution, and by constructing the distribution’s expected value and variance, it maximizes the utilization of sample data, thereby maintaining an equilibrium between data exploitation and the exploration of the unknown. Theoretical substantiation is provided to illustrate that the Bayesian regret associated with the algorithm exhibits sublinear growth. Empirical simulations corroborate that the algorithm in question outperforms traditional ε-greedy, Upper Confidence Bound (UCB), and Thompson sampling algorithms in terms of higher cumulative rewards, diminished total regret, accelerated convergence rates, and enhanced system throughput.

## 1. Introduction

The Space-Air-Ground Integrated Network (SAGIN) integrates satellite systems, aerial networks, and terrestrial communication infrastructures [1], thereby enabling global, uninterrupted coverage. It holds considerable potential for application in scenarios such as disaster response, intelligent transportation systems, and the evolution towards 6G communication networks [2]. The integration of cutting-edge technologies, including artificial intelligence, machine learning, and Software-Defined Networking (SDN), further augments the performance and adaptability of the SAGIN [3]. The pivotal technological hurdles encompass dynamic node management, interconnection of heterogeneous networks, resource allocation optimization, and the intelligent management of networks [4].

In the context of emergency communication, users are in constant motion, and terrestrial base stations may be insufficient to fulfill their communication demands, thus requiring the collaborative support of space and aerial networks [5]. To ensure the reliability of data transmission, it is imperative for users to swiftly connect to the network node at the highest rate within the signal range [6]. However, given the dynamic shifts in the location and status of network nodes, users must rapidly connect to the optimal network node guided by a specific algorithm, without the benefit of prior knowledge of the network conditions. Addressing how users in emergency communication scenarios can expeditiously access the most suitable network nodes has become an imperative issue.

## 2. Related Work

Online learning methodologies serve as efficacious algorithms for learning and forecasting within dynamic settings [7]. The Multi-Armed Bandit (MAB) problem, often referred to as the slot machine dilemma [8], is a quintessential issue in the realm of online learning. The MAB framework has garnered widespread application due to its capacity to facilitate access optimization even amidst a dearth of environmental information. In particular, ref. [9] investigates the selection process among multiple channels under the condition that channel information adheres to independent and identically distributed variations within a solitary user context, proposing a decision-making framework predicated on the Restless Multi-Armed Bandit (RMAB) model. Ref. [10] integrates the RMAB model, introducing a greedy algorithm for channel selection to augment the spectrum access efficiency for users. Ref. [11] pioneers the employment of index algorithms to address the archetypal MAB challenge, while ref. [12] refines the confidence parameter of the Upper Confidence Bound (UCB) algorithm to enhance its efficacy.

Existing studies on network selection are typically marred by two principal deficiencies: firstly, the oversight of the influence that immediate gains may exert on future earnings; secondly, the linear growth pattern of cumulative regret values yielded by current algorithms, which results in diminished learning efficiency and protracted convergence. Such outcomes are at odds with practical aspirations for achieving higher efficiency through straightforward methods.

Addressing these concerns, the present research introduces an enhanced index algorithm designed to refine the network selection process. This algorithm takes into account the interplay between immediate and prospective future gains, leveraging the strengths of both the UCB and Thompson sampling algorithms to achieve a harmonious equilibrium between exploration and exploitation. Simulation results indicate that, in contrast to extant methodologies, the dynamic variance sampling algorithm proposed herein not only escalates learning efficiency but also mitigates cumulative regret, thereby augmenting system throughput.

## 3. System Model

### 3.1. Network Architecture

**Figure 1:**
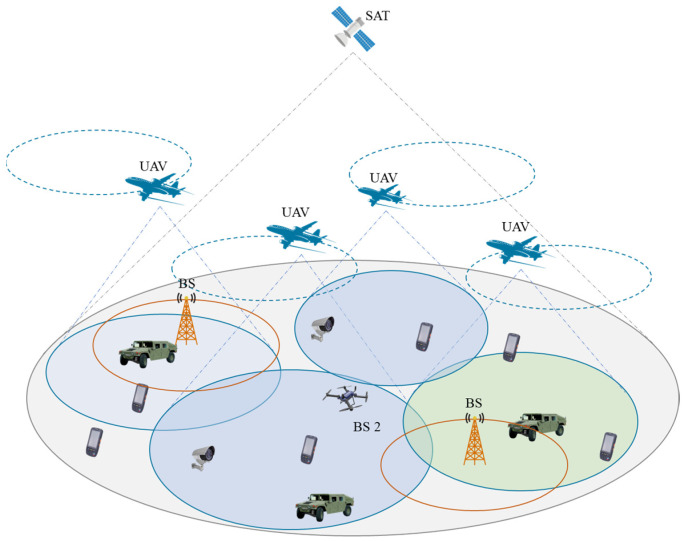
Network architecture.

The network architecture of this study is illustrated in Figure 1. The research area is conceptualized as a circular zone, equipped with satellites, unmanned aerial vehicles (UAVs), and base stations. The satellite signals ensure comprehensive coverage across the entire area, and UAVs operate on predefined circular trajectories, while the coverage of base stations is confined. Users are outfitted with multi-mode antennas capable of accessing multiple networks, yet they are restricted to connecting to a single network node at any given instant. The user communication is structured in a time-slotted manner, segmenting the user communication timeframe into T discrete time slots, with each slot separated by a relatively minor interval. The crux of this paper’s research lies in the decision-making process for an individual user to access the space–air–ground integrated emergency communication network node during each time slot.

### 3.2. Channel Model

The system includes three types of links: space-to-ground, aerial-to-ground, and base station-to-ground links. According to the literature [13], the channel state information (CSI) from the satellite to the user is as follows:

(1) $$h_{SAT}=\sqrt{C_{L}b\beta }\exp\left(-j\theta \right)$$

where $C_{L}$ denotes the free space loss, which can be calculated by the formula $C_{L}={\left(\lambda /4\pi \right)}^{2}/\left(d^{2}+l^{2}\right)$, where λ denotes the signal wavelength, l denotes the horizontal distance between the center of the satellite beam and the user, d denotes the vertical height of the satellite relative to the ground, β denotes the channel gain caused by the rain attenuation, which obeys the lognormal distribution, $\beta_{dB}$ is the β form of dB, θ is a phase vector uniformly distributed within the range [0,2π], and b indicates the satellite beam gain, which is defined as follows:

(2) $$b=G{\left(\frac{J_{1}\left(\mu_{0}\right)}{2\mu_{0}}-36\frac{J_{3}\left(\mu_{0}\right)}{{\mu_{0}}^{2}}\right)}^{2}$$

where G represents the maximum gain of the satellite antenna $\mu_{0}=2.07123\sin(\alpha )/\sin(\alpha_{3dB})$, α is the elevation angle between the center of the beam and the user, $\alpha_{3dB}$ is the 3 dB angle of the satellite beam, and $J_{1}(\cdot )$ and $J_{3}(\cdot )$ are the first-order and third-order Bessel functions of the first class, respectively.

According to the literature [13], the channel state information (CSI) from the aerial platform to the user is as follows:

(3) $$a_{UAV}=\sqrt{G_{L}}\left(\sqrt{\frac{K}{K+1}}a_{LoS}+\sqrt{\frac{1}{K+1}}a_{Ray}\right)$$

where $G_{L}=C_{0}/\left(U_{d}^{2}+U_{h}^{2}\right)$ denotes the path loss, where $C_{0}$ denotes the channel power gain for a reference distance of 1 m, $U_{d}$ is the horizontal distance from the UAV to the target user, and $U_{h}$ is the height of the UAV. The small-scale fading follows the Rice (Rician) channel model, where K is the Rician coefficient. $a_{LoS}$ is the line-of-sight Rician fading component and $a_{Ray}$ is the non-line-of-sight Rayleigh fading component.

According to the literature [13], the channel state information (CSI) from the base station to the user is as follows:

(4) $$g_{BS}=\sqrt{\alpha }g_{0}$$

where $\alpha =C_{0}r^{-4}$ represents the large-scale fading, where $C_{0}$ denotes the channel power gain at a reference distance of 1 m, r denotes the distance between the base station and the user, and *g*_0_ represents the small-scale fading, which follows a Nakagami-m distribution.

### 3.3. Communication Model

Based on the method of calculating the channel state information (CSI) mentioned, the communication rate that can be obtained by a user when connecting to a satellite, a UAV, and a base station, respectively (without considering inter-channel interference) can be expressed by the formula

(5) $$R_{SAT}=\log_{2}\left(1+\frac{p_{SAT}{\left|h_{SAT}\right|}^{2}}{{\delta_{SAT}}^{2}}\right)$$

(6) $$R_{UAV}=\log_{2}\left(1+\frac{p_{UAV}{\left|a_{UAV}\right|}^{2}}{{\delta_{UAV}}^{2}}\right)$$

(7) $$R_{BS}=\log_{2}\left(1+\frac{p_{BS}{\left|g_{BS}\right|}^{2}}{{\delta_{BS}}^{2}}\right)$$

In Equation (5), $p_{SAT}$ denotes the power of the satellite, $h_{SAT}$ denotes the CSI from the satellite to the user, and $\delta_{SAT}$ denotes the noise power of the satellite received by the user. Similarly, the meaning of each symbol in Equations (6) and (7) can be known.

### 3.4. Benefit Model

The benefit of the proposed algorithm in this paper after the user selects a network node to access is represented as in Equation (8):

(8) $$r=\frac{R_{node}}{R_{upper}}$$

where $R_{node}$ denotes the communication rate (i.e., $R_{SAT}$,$\;R_{UAV}$, or $R_{BS}$) that the user obtains from the network nodes, and $R_{upper}$ denotes the upper bound of the communication rate that the user is able to obtain from all the network nodes, e.g., when the maximal rate is 100, $R_{upper}\geq 100$, which ensures that the user’s gain interval is $\left[0,\;1\right]$.

Meanwhile, in order to experimentally compare the algorithm proposed in this paper with the traditional ε-greedy algorithm, UCB algorithm, and Thompson sampling algorithm, according to the literature [14], the gain r can be transformed into a Bernoulli random variable, and the current gain $r^{\prime }$ obeys the Bernoulli distribution with parameter r (i.e., $r^{\prime }\sim Bernoulli\left(r\right)$) with $r^{\prime }\in \left\{0,\;1\right\}$. The article has shown that the above two returns are algorithmically equivalent.

Furthermore, to experimentally compare the algorithm proposed in this paper with conventional algorithms, including the ε-greedy algorithm, the Upper Confidence Bound (UCB) algorithm, and the Thompson sampling algorithm, the reward r can be converted into a Bernoulli random variable, as outlined in reference [14]. The instantaneous reward $r^{\prime }$ is governed by a Bernoulli distribution parameterized by r (i.e., $r^{\prime }\sim Bernoulli\left(r\right)$) with $r^{\prime }\in \left\{0,\;1\right\}$. The aforementioned article has substantiated the algorithmic equivalence of these two forms of rewards.

### 3.5. Objective Function

The purpose of the algorithm based on the MAB model is to determine the user’s strategy for selecting a network node at each moment in time so as to maximize the expectation of the total gain over T time slots, i.e.,

(9) $$\max E\left[\sum_{t=1}^{T}r_{i\left(t\right)}\right]$$

where $i\left(t\right)$ denotes the network node i selected by the user according to the algorithm at moment t, and $r_{i\left(t\right)}$ denotes the gain after selecting network node i, corresponding to r and $r^{\prime }$ defined in the gain model. In order to compare the effects of different algorithms more intuitively, this paper uses the minimization of the expected total regret as the objective function corresponding to the maximization of the expected total gain, where the so-called regret is the expected rate lost at each moment due to the failure to select the best network node. Define $\mu_{i}$ as the expected gain of network node i, so that $\mu^{*}=\max\mu_{i}$ denotes the expected gain of the optimal network node, ${∆}_{i}=\mu^{*}-\mu_{i}$ denotes the suboptimal network gap, and also define $k_{i}\left(t\right)$ as the number of times that the network node i has been selected prior to the moment of t. Then the total regret of the user in *T* time slots is denoted as

(10) $$E\left[ℜ\left(T\right)\right]=E\left[\sum_{t=1}^{T}\left(\mu^{*}-\mu_{i\left(t\right)}\right)\right]=\sum_{i}\Delta_{i}\cdot E\left[k_{i}\left(T\right)\right]$$

## 4. Network Selection Mechanism Based on the MAB Model

### 4.1. Dynamic Variance Sampling Algorithm

The issue of user network node selection in an unknown environment has been modeled as a Multi-Armed Bandit (MAB) model. As an advanced dynamic stochastic control framework, the MAB model has excellent learning capabilities and is mainly used to solve problems of selection and resource allocation under limited resources. This includes, but is not limited to, scenarios such as channel allocation, opportunistic network access, and routing selection. Through the application of the MAB model, users can make optimal decisions in uncertain environments, thereby effectively enhancing the overall performance of the system. Reference [15] proposed an Upper Confidence Bound (UCB)-based index algorithm, which, although it reduces the algorithm complexity compared to traditional algorithms, still has a certain gap in overall revenue compared to the ideal state, and the convergence speed is slow; reference [16] studied the theoretical performance of the Thompson sampling algorithm, which, although it has a lower regret lower bound compared to the UCB index algorithm, still has a gap from the ideal state.

The index value of the UCB algorithm consists of two parts: the sample average reward of the current network and the confidence factor, which is represented as follows:

(11) $$\theta_{i}(t)={\hat{\mu }}_{i}\left(t\right)+\sqrt{2\ln\left(t-1\right)/k_{i}(t)}$$

where $k_{i}\left(t\right)$ denotes the number of times a network node has been selected at moment t, ${\hat{\mu }}_{i}\left(t\right)$ denotes the mean benefit of network node i before moment t, which reflects how well the algorithm utilizes the data, and the confidence factor $\sqrt{2\ln\left(t-1\right)/k_{i}\left(t\right)}$ is a quantity inversely related to the number of times it has been selected, which reflects how well the data has been explored.

The Thompson sampling algorithm is a stochastic algorithm based on Bayesian ideas, which selects arms at each time step based on their posterior probability of being the best arm by assuming a Beta prior distribution for the reward distribution parameter of each arm. The index values are calculated as follows:

(12) $$\theta_{i}(t)=\mathrm{Sample}[Beta(1+S_{i}\left(t\right),1+F_{i}\left(t\right)]$$

where $Sample\left[Beta\left(\cdot \right)\right]$ denotes the Beta distribution sampling, $S_{i}\left(t\right)$ and $F_{i}\left(t\right)$ denote the number of times that the network node has been selected with a gain of 1 and the number of times that the network node has been selected with a gain of 0 before the moment t, respectively.

The advantage of the UCB algorithm is the introduction of the confidence factor related to the number of times selected, which enhances the exploratory nature of the algorithm, and the disadvantage is the low exploratory efficiency and slow convergence speed; the advantages of the Thompson sampling algorithm are the introduction of the Bayesian sampling idea, that the assumption of the prior distribution is more in line with the actual scenario, and that its convergence speed has been improved, but it is a large gap with the ideal value. At the same time, from the CSI model, it can be seen that the changes in the state of network nodes are closer to the normal distribution, so the normal distribution is considered as the prior distribution of network state changes.

Based on the characteristics of the above two algorithms, this paper considers the improvement in terms of the advantages of the two algorithms. Bayesian sampling and the confidence factor are introduced into the algorithm at the same time, which assumes that the return of each network node obeys the normal distribution, the mean value of the sample is used as the expectation (reflecting the utilization of the data), the number of times of being selected is introduced into the sample variance (reflecting the exploration of the data), and the index value is calculated as follows:

(13) $$\theta_{i}(t)=Sample\left[N\left({\hat{\mu }}_{i}\left(t\right),\frac{1}{k_{i}(t)+1}\right)\right]$$

where Sample[N(·)] denotes the sampling of a normal distribution, ${\hat{\mu }}_{i}\left(t\right)$ denotes the mean benefit of network node i before time t, and $k_{i}\left(t\right)$ denotes the number of times network node i is selected at time t. The update rule is as follows:

(14) $${\hat{\mu }}_{i}(t+1)=\left\{ \begin{array}{l} \frac{{\hat{\mu }}_{i}(t)\times k_{i}(t)+r_{i}(t)}{k_{i}(t)+1},\;select\;i\\ {\hat{\mu }}_{i}(t)\;\;\;\;\;\;\;\;\;\;\;\;,\;other \end{array}\right.$$

(15) $$k_{i}(t+1)=\left\{ \begin{array}{l} k_{i}(t)+1\;,\;select\;i\\ k_{i}(t)\;\;\;\;\;\;\;\;,\;other \end{array}\right.$$

### 4.2. Theoretical Analysis and Proof

**Definition** **1.** $k_{i}\left(t\right)$ *denotes the number of times network node i has been selected prior to moment t. $S_{i}\left(t\right)$ and $F_{i}\left(t\right)$ denote the number of times network node i has been selected prior to moment t with a gain of 1 and a gain of 0, respectively. $i\left(t\right)$ denotes the value of the index of the network node that has been selected at moment t.*

**Definition** **2.** *Assuming that network node 1 is the optimal network and that $\mu_{i}$ denotes the expected return of network node i, there is $\mu_{i}<\mu_{1}$ for ∀i≠1. Define $x_{i}$, $y_{i}$ as two real numbers that satisfy $\mu_{i}<x_{i}<y_{i}<\mu_{1}$, and obviously $x_{i}$ and $y_{i}$ must exist. Define $L_{i}\left(T\right)=lnT/d(x_{i},y_{i})$, where $d\left(x_{i},y_{i}\right)=x_{i}\ln\left(x_{i}/y_{i}\right)+(1-x_{i})ln[\left(1-x_{i}\right)/\left(1-y_{i}\right)]$ denotes the KL dispersion between Bernoulli distributions with the parameters $x_{i}$ and $y_{i}$, respectively, of the KL distance.*

**Definition** **3.** $\theta_{i}\left(t\right)$ *denotes the sample values sampled by the algorithm from the posterior distribution of network node* i *at moment* t *and* $\theta_{i}\left(t\right)\sim N\left({\hat{\mu }}_{i}\left(t\right),1/\left(k_{i}\left(t\right)+1\right)\right)$.

**Definition** **4.** ${\hat{\mu }}_{i}\left(t\right)$ *denotes the mean value of the returns of network node i at moment t, defined as ${\hat{\mu }}_{i}\left(t\right)=\sum_{\tau =1,i\left(\tau \right)=i}^{t-1}r_{i}(\tau )/\left[k_{i}\left(t\right)+1\right]$, for i≠1, defining $E_{i}^{\mu }\left(t\right)$ as the event ${\hat{\mu }}_{i}\left(t\right)\leq x_{i}$, and $E_{i}^{\theta }\left(t\right)$ as the event $\theta_{i}\left(t\right)\leq y_{i}$.*

It can be seen that both ${\hat{\mu }}_{i}\left(t\right)$ and $\theta_{i}\left(t\right)$ are approximations of the true expected return of network node i. The former is an empirical estimate, the latter is a sampled sample from the posterior distribution, and $x_{i}$ and $y_{i}$ are upper bounds on the true return expectation of network node i. Thus, intuitively, the significance of $E_{i}^{\mu }\left(t\right)$ and $E_{i}^{\theta }\left(t\right)$ is that these two estimates should not be overestimated too much; specifically, do not exceed the thresholds $x_{i}$ and $y_{i}$.

**Definition** **5.** $F_{t}$ *denotes the sequence of historical policy information prior to moment t, defined as $F_{t}=\left\{i\left(\tau \right),r_{i(\tau )},\tau =1,\ldots ,t\right\}$, where $i\left(\tau \right)$ denotes the index value of the network node that is selected at moment τ, and $r_{i(\tau )}$ is its corresponding gain. Defining $F_{0}=\{\}$ and satisfying $F_{0}\subseteq F_{1}\subseteq \ldots \subseteq F_{T-1}$, it can be seen that the distributions of $k_{i}\left(t\right)$, ${\hat{\mu }}_{i}\left(t\right)$, and $\theta_{i}\left(t\right)$ in the above definitions, and whether or not the events $E_{i}^{\mu }\left(t\right)$ and $E_{i}^{\theta }\left(t\right)$ occur, are all determined by $F_{t-1}$.*

**Definition** **6.** *Defining $p_{i,t}=P\left(\theta_{1}\left(t\right)>y_{i}|F_{t-1}\right)$, it can be seen that $p_{i,t}$ is a random variable determined by $F_{t-1}$.*

**Lemma** **1.** *According to Lemma 1 of reference* [17]*, when * i≠1 *, we have the following for any* t *and* $F_{t-1}$ *:*

(16) $$P\left(i\left(t\right)=i,E_{i}^{\mu }\left(t\right),E_{i}^{\theta }\left(t\right)|{ℱ}_{t-1}\right)\leq \frac{1-p_{i,t}}{p_{i,t}}P\left(i\left(t\right)=1,E_{i}^{\mu }\left(t\right),E_{i}^{\theta }\left(t\right)|{ℱ}_{t-1}\right)$$

**Lemma** **2.** *Let* $\tau_{j}$ *denote the moment when network node 1 is selected for the*  j*th time, which can be obtained according to Lemma 2.13 of reference* [18]:

(17) $$\sum_{j=0}^{T-1}E\left[\frac{1}{p_{i,\tau_{j}+1}}-1\right]\leq \left\{ \begin{array}{l} e^{11}+5,\;\forall j\\ \frac{4}{T\Delta_{i}^{2}}\;\;\;\;,j>4L_{i}(T) \end{array}\right.$$

 *where* $L_{i}\left(T\right)=18\ln\left(T{∆}_{i}^{2}\right)/{∆}_{i}^{2}$.

**Fact** **1** (Chernoff–Hoeffding Bound)**.** *Let $X_{1},\ldots ,X_{n}$ be random variables on the interval [0, 1] and $E\left(X_{t}|X_{1},\ldots ,X_{t-1}\right)=\mu$, so that $S_{n}=X_{1}+\cdots +X_{n}$, and there is the following for any a≥0:*

(18) $$P\left(S_{n}\geq n\mu +a\right)\leq e^{-2a^{2}/n}$$

(19) $$P\left(S_{n}\leq n\mu -a\right)\leq e^{-2a^{2}/n}$$

**Fact** **2.** *Let Z denote a random variable obeying a normal distribution with mean μ and variance $\sigma^{2}$ for ∀z∈Z with*

(20) $$\frac{1}{4\sqrt{\pi }}\cdot e^{-7z^{2}/2}\leq P\left(\left|Z-\mu \right|>z\sigma \right)\leq \frac{1}{2}e^{-z^{2}/2}$$

**Theorem** **1.** *The upper bound on the regret of the dynamic variance sampling algorithm is given by*

(21) $$E\left[ℜ\left(T\right)\right]\leq O(\sqrt{NT\ln N})$$

 *where* T *is the total time duration and* N *is the number of network nodes*.

**Proof.**  According to the definition of regret in Equation (10), we can see that

$$E\left[ℜ\left(T\right)\right]=\sum_{i}\Delta_{i}\cdot E\left[k_{i}\left(T\right)\right]$$

Firstly, the regret is decomposed according to the events defined in Definition 4, but here instead of decomposing the regret directly, the expectation of the number of times a suboptimal network node is selected is decomposed. Because according to the definition of regret, the expectation of the number of times a network node is selected is multiplied by the suboptimal network gap and summed up to get the final regret, and the optimal network node does not contribute to the regret, it is only necessary to decompose the number of times a suboptimal network node is selected. For ∀i≠1, there is

$$E\left[k_{i}\left(T\right)\right]=E\left[\sum_{t=1}^{T}I\left(i\left(t\right)=i\right)\right]=\sum_{t=1}^{T}E\left[I\left(i\left(t\right)=i\right)\right]=\sum_{t=1}^{T}\left(i\left(t\right)=i\right)$$

where I(·) denotes the indicator function, and the decomposition of the above equation using the event $E_{i}^{\mu }\left(t\right)$ and its complement $\bar{E_{i}^{\mu }\left(t\right)}$ is obtained:

$$E\left[k_{i}\left(T\right)\right]=\sum_{t=1}^{T}P\left(i\left(t\right)=i,E_{i}^{\mu }\left(t\right)\right)+\sum_{t=1}^{T}P\left(i\left(t\right)=i,\bar{E_{i}^{\mu }\left(t\right)}\right)$$

Continuing to decompose the above equation by the event $E_{i}^{\theta }\left(t\right)$ yields

(22) $$\begin{array}{l} E\left[k_{i}\left(T\right)\right]=\underset{①}{\underset{︸}{\sum_{t=1}^{T}P\left(i\left(t\right)=i,E_{i}^{\theta }\left(t\right),E_{i}^{\mu }\left(t\right)\right)}}+\underset{②}{\underset{︸}{\sum_{t=1}^{T}P\left(i\left(t\right)=i,\bar{E_{i}^{\theta }\left(t\right)},E_{i}^{\mu }\left(t\right)\right)}}\\ \;\;\;\;\;\;\;\;\;\;\;\;\;\;\;\;\;\;+\underset{③}{\underset{︸}{\sum_{t=1}^{T}P\left(i\left(t\right)=i,\bar{E_{i}^{\mu }\left(t\right)}\right)}} \end{array}$$

Next, derive the upper bounds for each of the above three terms, starting with the upper bound for Equation (1). Combined with the relationship stated in Definition 5, this is obtained from the properties of conditional probability and Lemma 1:

(23) $$\begin{array}{ll} & \sum_{t=1}^{T}P\left(i\left(t\right)=i,E_{i}^{\theta }\left(t\right),E_{i}^{\mu }\left(t\right)\right)\\ = & \sum_{t=1}^{T}E\left[P\left(i\left(t\right)=i,E_{i}^{\theta }\left(t\right),E_{i}^{\mu }\left(t\right)|{ℱ}_{t-1}\right)\right]\\ \leq & \sum_{t=1}^{T}E\left[\frac{1-p_{i,t}}{p_{i,t}}P(i\left(t\right)=1,E_{i}^{\theta }\left(t\right),E_{i}^{\mu }\left(t\right)|{ℱ}_{t-1})\right]\\ = & \sum_{t=1}^{T}E\left[E\left[\frac{1-p_{i,t}}{p_{i,t}}I\left(i\left(t\right)=1,E_{i}^{\theta }\left(t\right),E_{i}^{\mu }\left(t\right)|{ℱ}_{t-1}\right)\right]\right]\\ = & \sum_{t=1}^{T}E\left[\frac{1-p_{i,t}}{p_{i,t}}I\left(i\left(t\right)=1,E_{i}^{\theta }\left(t\right),E_{i}^{\mu }\left(t\right)\right)\right] \end{array}$$

The second equality sign above utilizes the property that $p_{i,t}$ is invariant given $F_{t-1}$. Combined with $p_{i,t}=P\left(\theta_{1}\left(t\right)>y_{i}|F_{t-1}\right)$, it can be seen that the value of $p_{i,t}$ only changes with the distribution of $\theta_{1}\left(t\right)$, i.e., only after network node 1 is selected. Defining $\tau_{j}$ to denote the moment when network node 1 is selected for the jth time, the values of $p_{i,t}$ are equal at all $t\in \left\{\tau_{j}+1,\ldots ,\tau_{j+1}\right\}$ moments. Therefore,

(24) $$\begin{array}{ll} & \sum_{t=1}^{T}E\left[\frac{1-p_{i,t}}{p_{i,t}}I\left(i\left(t\right)=1,E_{i}^{\theta }\left(t\right),E_{i}^{\mu }\left(t\right)\right)\right]\\ = & \sum_{j=1}^{T-1}E\left[\frac{1-p_{i,\tau_{j}+1}}{p_{i,\tau_{j}+1}}\sum_{t=\tau_{j}+1}^{\tau_{j+1}}I\left(i\left(t\right)=1,E_{i}^{\theta }\left(t\right),E_{i}^{\mu }\left(t\right)\right)\right]\\ \leq & \sum_{j=0}^{T-1}E\left[\frac{1-p_{i,\tau_{j}+1}}{p_{i,\tau_{j}+1}}\right] \end{array}$$

Let $L_{i}\left(T\right)=18\ln\left(T{∆}_{i}^{2}\right)/{∆}_{i}^{2}$, and by Lemma 2, when $j\geq 4L_{i}$, there are

(25) $$E\left[\frac{1-p_{i,\tau_{j}+1}}{p_{i,\tau_{j}+1}}\right]\leq \frac{4}{T\Delta_{i}^{2}}$$

Substituting Equation (25) into Equation (24) gives the upper bound of Equation (1):

(26) $$\sum_{t=1}^{T}P\left(i\left(t\right)=i,E_{i}^{\theta }\left(t\right),E_{i}^{\mu }\left(t\right)\right)\leq 4L_{i}\left(T\right)\left(e^{64}+4\right)+\frac{4}{\Delta_{i}^{2}}$$

Next, the upper bound of Equation (2) is derived, and Equation (2) is decomposed into two parts according to the magnitude relationship between $k_{i}(t)$ and $L_{i}\left(T\right)$:

(27) $$\begin{array}{ll} \sum_{t=1}^{T}P\left(i\left(t\right)=i,\bar{E_{i}^{\theta }\left(t\right)},E_{i}^{\mu }\left(t\right)\right) & =\sum_{t=1}^{T}P\left(i\left(t\right)=i,k_{i}\left(t\right)\leq L_{i}\left(T\right),\bar{E_{i}^{\theta }\left(t\right)},E_{i}^{\mu }\left(t\right)\right)\\ & \;+\sum_{t=1}^{T}P\left(i\left(t\right)=i,k_{i}\left(t\right)>L_{i}\left(T\right),\bar{E_{i}^{\theta }\left(t\right)},E_{i}^{\mu }\left(t\right)\right) \end{array}$$

First analyzing the first term of Equation (27),we can get

(28) $$\begin{array}{l} \sum_{t=1}^{T}P\left(i\left(t\right)=i,k_{i}\left(t\right)\leq L_{i}\left(T\right),\bar{E_{i}^{\theta }\left(t\right)},E_{i}^{\mu }\left(t\right)\right)\\ \leq E\left[\sum_{t=1}^{T}I\left(i\left(t\right)=i,k_{i}\left(t\right)\leq L_{i}\left(T\right)\right)\right]\\ \leq L_{i}\left(T\right) \end{array}$$

Next, analyzing the latter term of Equation (27), if the value of $k_{i}(t)$ is large and the event $E_{i}^{\mu }\left(t\right)$ occurs, then the probability of the event $\bar{E_{i}^{\theta }\left(t\right)}$ occurring will be very small, and in conjunction with the definition of the event in Definition 4, there is

(29) $$\begin{array}{ll} & \sum_{t=1}^{T}P\left(i\left(t\right)=i,k_{i}\left(t\right)>L_{i}\left(T\right),\bar{E_{i}^{\theta }\left(t\right)},E_{i}^{\mu }\left(t\right)\right)\\ \leq & E\left[\sum_{t=1}^{T}P\left(i\left(t\right)=i,\bar{E_{i}^{\theta }\left(t\right)}|k_{i}\left(t\right)>L_{i}\left(T\right),E_{i}^{\mu }\left(t\right),{ℱ}_{t-1}\right)\right]\\ \leq & E\left[\sum_{t=1}^{T}P\left(\theta_{i}\left(t\right)>y_{i}|k_{i}\left(t\right)>L_{i}\left(T\right),{\hat{\mu }}_{i}\left(t\right)\leq x_{i},{ℱ}_{t-1}\right)\right] \end{array}$$

From the definition, we can see that $\theta_{i}(t)\sim N\left({\hat{\mu }}_{i}\left(t\right),1/\left(k_{i}\left(t\right)+1\right)\right)$, and according to the normal distribution property, given ${\hat{\mu }}_{i}\left(t\right)<x_{i}$, there are

(30) $$P[Sample\left[N\left({\hat{\mu }}_{i}\left(t\right),\sigma^{2}\right)\right]]\leq P[Sample\left[N\left(x_{i},\sigma^{2}\right)\right]]$$

Therefore, Equation (29) can be further scaled as

(31) $$\begin{array}{ll} & E\left[\sum_{t=1}^{T}P\left(\theta_{i}\left(t\right)>y_{i}|k_{i}\left(t\right)>L_{i}\left(T\right),{\hat{\mu }}_{i}\left(t\right)\leq x_{i},{ℱ}_{t-1}\right)\right]\\ \leq & E\left[\sum_{t=1}^{T}P\left(Sample\left[N\left(x_{i},\frac{1}{k_{i}\left(t\right)+1}\right)>y_{i}\right]|k_{i}\left(t\right)>L_{i}\left(T\right),{ℱ}_{t-1}\right)\right] \end{array}$$

From the probability density function of normal distribution and its distribution characteristics, when $k_{i}(t){>L}_{i}\left(T\right)$, there are

(32) $$P\left(Sample\left[N\left(x_{i},\frac{1}{k_{i}\left(t\right)+1}\right)>y_{i}\right]\right)\leq \frac{1}{2}e^{-\frac{\left(k_{i}\left(t\right)+1\right){\left(y_{i}-x_{i}\right)}^{2}}{2}}\leq \frac{1}{2}e^{-\frac{L_{i}\left(t\right)\cdot {\left(y_{i}-x_{i}\right)}^{2}}{2}}$$

Taking $x_{i}=\mu_{i}+{∆}_{i}/3$ and $y_{i}=\mu_{1}-{∆}_{i}/3$, $y_{i}-x_{i}={∆}_{i}/3$ and substituting in Equation (32):

(33) $$P(Sample\left[N\left(x_{i},\frac{1}{k_{i}\left(t\right)+1}\right)>y_{i}\right])\leq \frac{1}{2}e^{-\frac{L_{i}\left(t\right)\cdot {\left(y_{i}-x_{i}\right)}^{2}}{2}}=\frac{1}{2T\Delta_{i}^{2}}$$

Therefore,

(34) $$\sum_{t=1}^{T}P\left(i\left(t\right)=i,k_{i}\left(t\right)>L_{i}\left(T\right),\bar{E_{i}^{\theta }\left(t\right)},E_{i}^{\mu }\left(t\right)\right)\leq \frac{1}{2T\Delta_{i}^{2}}$$

Substituting Equations (28) and (34) into Equation (27) gives the upper bound of Equation (2) as

(35) $$\sum_{t=1}^{T}P\left(i\left(t\right)=i,\bar{E_{i}^{\theta }\left(t\right)},E_{i}^{\mu }\left(t\right)\right)\leq L_{i}\left(T\right)+\frac{1}{2T\Delta_{i}^{2}}$$

Finally, to derive the upper bound of Equation (3), define $\tau_{k}$ to denote the moment of the kth selection of network node i. Let $\tau_{k}=0$. According to the definition of $E_{i}^{\mu }\left(t\right)$, we can see that $\bar{E_{i}^{\mu }\left(t\right)}$ denotes the event ${\hat{\mu }}_{i}\left(t\right)>x_{i}$, and then we have

(36) $$\sum_{t=1}^{T}P\left(i\left(t\right)=i,\bar{E_{i}^{\mu }\left(t\right)}\right)\leq \sum_{k=0}^{T-1}P\left(\bar{E_{i}^{\mu }\left(\tau_{k+1}\right)}\right)$$

When k≥1, there is

(37) $${\hat{\mu }}_{i}\left(\tau_{k+1}\right)=\frac{S_{i}\left(\tau_{k+1}\right)}{k+1}\leq \frac{S_{i}\left(\tau_{k+1}\right)}{k}$$

According to the Chernoff–Hoeffding Bound (Fact 2):

(38) $$P\left(\bar{E_{i}^{\mu }\left(\tau_{k+1}\right)}\right)=P\left({\hat{\mu }}_{i}\left(\tau_{k+1}\right)>x_{i}\right)\leq P\left(\frac{S_{i}\left(\tau_{k+1}\right)}{k}>x_{i}\right)\leq e^{-kd\left(x_{i},\mu_{i}\right)}$$

Substitute Equation (38) into Equation (36):

(39) $$\sum_{t=1}^{T}P\left(i\left(t\right)=i,\bar{E_{i}^{\mu }\left(t\right)}\right)\leq \sum_{k=1}^{T-1}e^{-kd\left(x_{i},\mu_{i}\right)}\leq 1+\frac{1}{d\left(x_{i},\mu_{i}\right)}$$

Taking $x_{i}=\mu_{i}+{∆}_{i}/3$, we have by Pinsker’s inequality:

(40) $$d\left(x_{i},\mu_{i}\right)\geq 2{\left(x_{i}-\mu_{i}\right)}^{2}=\frac{2\Delta_{i}^{2}}{9}$$

Substituting Equations (26), (35), and (41) into Equation (22), respectively, yields

(41) $$\begin{array}{ll} E\left[k_{i}\left(T\right)\right] & \leq 4L_{i}\left(T\right)\left(e^{64}+4\right)+\frac{4}{\Delta_{i}^{2}}+L_{i}\left(T\right)+\frac{1}{2T\Delta_{i}^{2}}+1+\frac{9}{2\Delta_{i}^{2}}\\ & \leq \frac{72\ln\left(T\Delta_{i}^{2}\right)}{\Delta_{i}^{2}}\left(e^{64}+4\right)+\frac{4}{\Delta_{i}^{2}}+\frac{18\ln\left(T\Delta_{i}^{2}\right)}{\Delta_{i}^{2}}+\frac{1}{2\Delta_{i}^{2}}+1+\frac{9}{2\Delta_{i}^{2}} \end{array}$$

Therefore, the expectation of the regret upper bound for network node i is

(42) $$\Delta_{i}E\left[k_{i}\left(T\right)\right]\leq \frac{72\ln\left(T\Delta_{i}^{2}\right)}{\Delta_{i}}\left(e^{64}+5\right)+\Delta_{i}+\frac{9}{\Delta_{i}}$$

The value of the above equation decreases with ${∆}_{i}$ when ${∆}_{i}\geq e/\sqrt{T}$, and the upper bound of the expected regret is when ${∆}_{i}\geq e\sqrt{NlnN/T}$ for all network nodes is

$$O\left(\sqrt{\frac{T\ln N}{N}}+1\right)$$

When ${∆}_{i}\leq e/\sqrt{T}$, the upper bound on total regret is

(43) $$E\left[ℜ\left(T\right)\right]=e\sqrt{NT\ln N}=O\left(\sqrt{NT\ln N}\right)$$

□

The proof is complete. This theorem shows that the algorithm proposed in this paper is able to make the system regret value grow sublinearly and eventually converge, which is better than the algorithm with linear growth and can improve the system throughput. The notation and terminology involved in the proof are recapitulated in Table 1.

### 4.3. Algorithm Description and Procedure

Based on the above analysis, the algorithm based on the MAB model always selects the network node access corresponding to the maximum value of the current time-slot index, so that the algorithm proceeds along the direction of minimizing the total regret value. Users can explore the node with the optimal network state in a short time by using the algorithm proposed in this paper under the condition of an unknown network state. The steps of the algorithm are as follows (Algorithm 1):
**Algorithm 1.** Dynamic variance sampling algorithm flow.for t=1,2,⋯,T do        for each node i=1,…,N, sample $\theta_{i}(t)$ independently from the $N\left({\hat{\mu }}_{i}\left(t\right),\frac{1}{k_{i}\left(t\right)+1}\right)$ distribution        select node: $i(t)={argmax}_{i}\theta_{i}(t)$
        observe reward: $r_{i(t)}$
        update selected times: $k_{i(t)}\left(t+1\right)=k_{i(t)}\left(t\right)+1$        update mean benefit: $\mu_{i\left(t\right)}(t+1)=\frac{\mu_{i\left(t\right)}\left(t\right)\times k_{i(t)}\left(t\right)+r_{i(t)}}{k_{i(t)}+1}$ end for

## 5. Performance and Evaluation

In this paper, we consider the problem of how users choose the optimal network node access under an unknown network environment, and adopt a dynamic variance sampling algorithm to explore and learn the unknown network environment, and choose the optimal network node access based on historical experience prediction. This subsection simulates and analyzes the algorithm from the perspective of its performance parameters by establishing a simulation scenario, and compares its performance with the traditional ε-greedy algorithm, UCB algorithm, and Thompson sampling algorithm, respectively.

### 5.1. Simulation Settings

According to the relevant formulas in the channel model, communication model, and revenue model, the meanings of the relevant symbols and the simulation parameter settings are shown in Table 2.

The parameter settings related to the simulation scenario are shown in Table 3.

**Figure 2:**
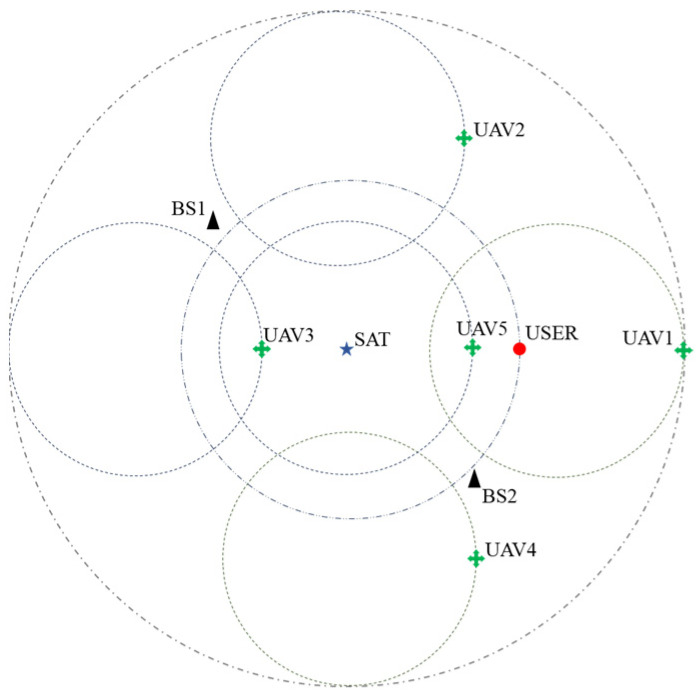
Schematic diagram of the simulation scene.

The scene schematic is shown in Figure 2. The reference coordinate system is the polar coordinate system with the center of the scene as the coordinate origin, the satellite is located at the origin of the coordinate system (0, 0), the coordinates of the flight centers of the UAVs are set to be $\left(250,\;0\right),\;\;\left(250,\;\;\pi /2\right),\;\;\left(250,\;\;\pi \right),\;\;\left(250,-\pi /2\right),\;\;(0,\;0)$, the flight altitudes are set to be 200, 225, 250, 275, 300, respectively, the initial positions are all set to be on the positive half-axis of the *x*-axis with the respective flight centers as the origin, and the positions of the base stations are set to be $\left(200,\;3\pi /2\right),\;\;\left(200,\;-\pi /2\right)$, respectively. The coordinates of the user’s motion center are (0, 0) and the initial position is set to (200, 0). The total time slot of the simulation is set to be 1000, and in order to overcome the randomness of the network environment, each group of experiments is repeated 100 times.

### 5.2. Results and Analysis

**Figure 3:**
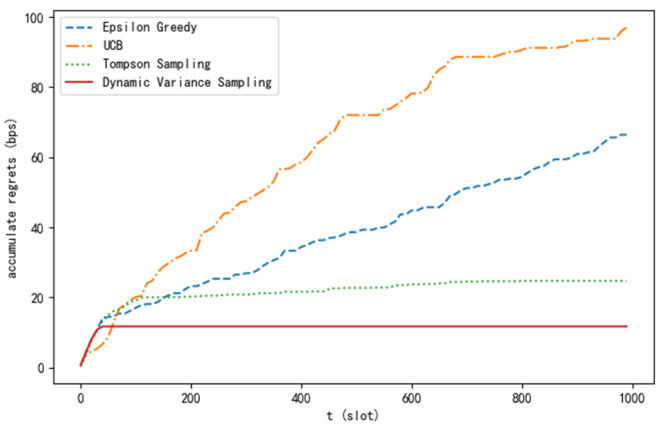
Cumulative regret curve of different algorithms.

The experiment compares the dynamic variance sampling algorithm proposed in this paper with current mainstream algorithms (ε-greedy [18], UCB, and Thompson sampling, etc.) from different perspectives, ultimately verifying the superiority of this algorithm. As can be seen from Figure 3, the cumulative regret value of the algorithm proposed in this paper grows in an approximately logarithmic relationship with the time slot and ultimately converges, which confirms the deduction of Theorem 1.

**Figure 4:**
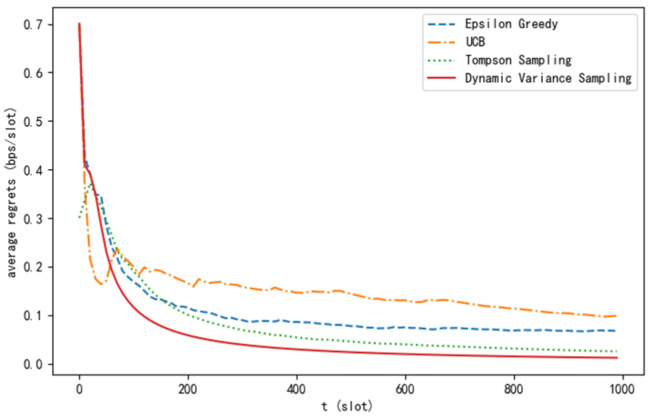
Average regret curve of different algorithms.

From Figure 4, it can be observed that as the number of time slots increases, all four algorithms are capable of gradually reducing the average regret value towards stability. However, the dynamic variance sampling algorithm proposed in this paper has a faster convergence rate compared to the other three algorithms, and the average regret value approaches closer to 0.

**Figure 5:**
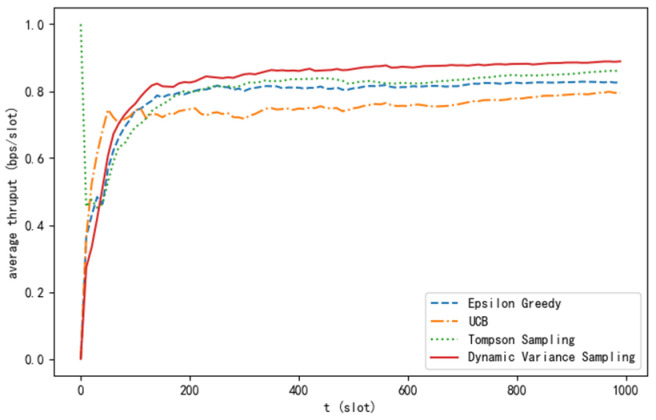
Average throughput curve of different algorithms.

Figure 5 presents the comparison results of the average throughput for the four algorithms. It can be seen that the average throughput of the algorithm proposed in this paper is significantly higher than that of the other algorithms. When the number of time slots is sufficiently large, the average throughput approaches the ideal value. 

**Figure 6:**
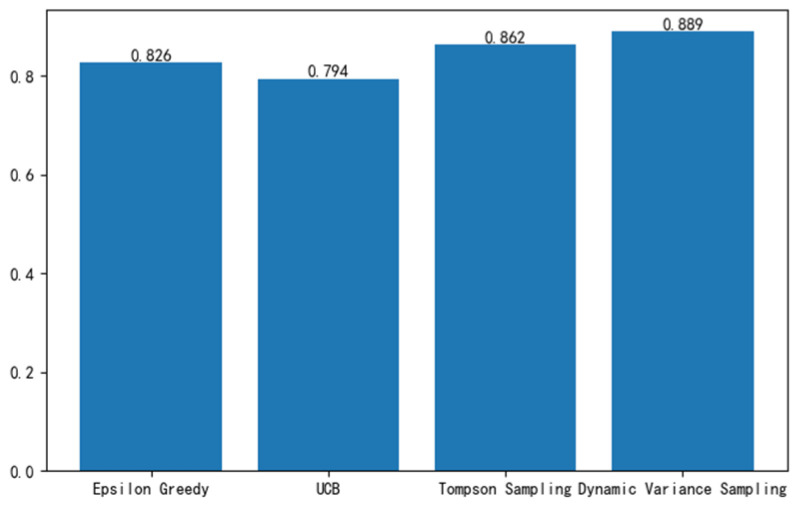
Average throughput after 1000 iterations.

Figure 6 illustrates the mean throughput of the four algorithms following 1000 iterations. Analysis of the figure reveals that, within the simulated environment described, the mean throughput of the algorithm introduced in this study has experienced enhancements of 7.63%, 11.96%, and 3.13% relative to the ε-greedy algorithm, Upper Confidence Bound (UCB) algorithm, and Thompson sampling algorithm, respectively.

## 6. Conclusions

This study, predicated on the MAB model, explores the dynamic selection of and access to network nodes by users within an integrated space–air–ground emergency communication network, given the condition of uncertain node network states. Comparative simulation experiments were conducted across various online learning algorithms. The ε-greedy algorithm, rooted in a greedy heuristic, prioritizes immediate gains; the Upper Confidence Bound (UCB) algorithm focuses on static assessments and lacks consideration of the interplay between sequential strategies, leading to reduced adaptability; the Thompson sampling algorithm, conversely, offers more consistent outcomes. The simulation outcomes underscore that the algorithm introduced in this paper adeptly harmonizes the interplay between exploration and exploitation inherent in network node states. In scenarios where the network node state information is obfuscated, the algorithm proposed herein markedly diminishes the learning regret, expedites the convergence pace, and augments system throughput. Nonetheless, the current research is primarily concentrated on the network node access mechanism for individual users, and further investigation is warranted to address the network node access mechanisms for multiple users within the context of an integrated space–air–ground emergency communication network.

## Figures and Tables

**Table 1:** Review of the main symbols.

Symbol	Meaning of Symbol
$k_{i}\left(t\right)$	The number of times network node i has been selected before moment t
$S_{i}\left(t\right)$	The number of times network node i has gained 1 after being selected before moment t
$F_{i}\left(t\right)$	The number of times network node i has gained 0 after being selected before moment t
$i\left(t\right)$	Index of the selected network node at moment t
$r_{i\left(t\right)}$	Benefit of network node i after being selected at moment t
$\mu_{i}$	Expected benefit of network node i
${∆}_{i}$	$\mu_{1}-\mu_{i}$
$x_{i},y_{i}$	Real numbers satisfying $\mu_{i}<x_{i}<y_{i}<\mu_{1}$
$d\left(x_{i},y_{i}\right)$	The KL dispersion between the Bernoulli distributions of $x_{i}$ and $y_{i}$
T	Total number of time slots
τ	Variables in the definition of Ft that depend on the value taken at time *t*
$L_{i}\left(T\right)$	$lnT/d(x_{i},y_{i})$
$\theta_{i}\left(t\right)$	Sample values sampled by the algorithm in the posterior distribution of network node *i* at moment *t*
${\hat{\mu }}_{i}\left(t\right)$	The mean value of the benefit of network node i at time t
$E_{i}^{\mu }\left(t\right)$	Event ${\hat{\mu }}_{i}\left(t\right)\leq x_{i}$
$E_{i}^{\theta }\left(t\right)$	Event $\theta_{i}\left(t\right)\leq y_{i}$
$F_{t}$	Sequence of historical strategy information up to moment t
$p_{i,t}$	$P\left(\theta_{1}\left(t\right)>y_{i}|F_{t-1}\right)$
P(·)	Probability of an event
E(·)	Expectation calculus
$I\left(\cdot \right)$	Indicator function
$N\left(\mu ,\sigma^{2}\right)$	Normal distribution
$Sample\left[\cdot \right]$	Sampling the distribution

**Table 2:** Meanings of symbols and simulation parameter settings.

Symbol	Meaning of Symbol	Value
d	Satellite orbital altitude	600 km
l	Horizontal distance between satellite beam center and user	0
$\beta_{dB}$	Satellite channel gain caused by rain attenuation	$ln(\beta_{dB})\sim N(-3.125,1.6)$
G	Maximum gain of satellite antenna	52 dB
$\alpha_{3dB}$	3 dB angle of satellite beam	0.4
$p_{SAT}$	Satellite downlink power	120 dBW
$U_{d}$	Horizontal distance from UAV to user	0~400 m
$U_{h}$	The height of the drone	200~300 m
K	Rician coefficient of UAV small-scale fading	10 dB
$p_{UAV}$	Downlink power of UAV	3 dBW
$C_{0}$	Channel power gain with a reference distance of 1 m	40 dB
r	Distance between base station and user	0~400 m
$p_{BS}$	Downlink power of base station	20 dBW
$g_{0}$	Small-scale fading of base station	$g_{0}\sim Nakagami-m(2,1)$
$\delta_{SAT},\delta_{UAV},\delta_{BS}$	Noise power received by users	1 dBW
$R_{upper}$	Upper bound of communication rate	100

**Table 3:** Scene parameter settings.

Parameter	Value
Scene radius	400 m
Number of satellites	1
Number of base stations	2
Number of UAVs	5
Angular velocity of UAV	5°/s
Flight radius of UAV	150 m
User moving radius	200 m
User angular velocity	0.5°/s

## Data Availability

Data are contained within the article.
